# Development and validation of a 3D‐printed bolus cap for total scalp irradiation

**DOI:** 10.1002/acm2.12552

**Published:** 2019-03-01

**Authors:** Garrett C. Baltz, Pai‐Chun Melinda Chi, Pei‐Fong Wong, Congjun Wang, Daniel F. Craft, Stephen F. Kry, Stacy Sydney Hsinyi Lin, Adam S. Garden, Susan A. Smith, Rebecca M. Howell

**Affiliations:** ^1^ Department of Radiation Physics The University of Texas MD Anderson Cancer Center Houston TX USA; ^2^ Medical Physics Program The University of Texas MD Anderson Cancer Center UTHealth Graduate School of Biomedical Sciences Houston TX USA; ^3^ Department of Radiation Oncology The University of Texas MD Anderson Cancer Center Houston TX USA

**Keywords:** 3D printing, bolus, dosimetry, total scalp irradiation

## Abstract

**Purpose:**

The goal of total scalp irradiation (TSI) is to deliver a uniform dose to the scalp, which requires the use of a bolus cap. Most current methods for fabricating bolus caps are laborious, yet still result in nonconformity and low reproducibility, which can lead to nonuniform irradiation of the scalp. We developed and validated patient‐specific bolus caps for TSI using three‐dimensional (3D) printing.

**Methods and materials:**

3D‐printing materials were radiologically analyzed to identify a material with properties suitable for use as a bolus cap. A Python script was developed within a commercial treatment planning system to automate the creation of a ready‐to‐print, patient‐specific 3D bolus cap model. A bolus cap was printed for an anthropomorphic head phantom using a commercial vendor and a computed tomography simulation of the anthropomorphic head phantom and bolus cap was used to create a volumetric‐modulated arc therapy TSI treatment plan. The planned treatment was delivered to the head phantom and dosimetric validation was performed using thermoluminescent dosimeters (TLD). The developed procedure was used to create a bolus cap for a clinical TSI patient, and *in vivo* TLD measurements were acquired for several fractions.

**Results:**

Agilus‐60 was validated as a new 3D‐printing material suitable for use as bolus. A 3D‐printed Agilus‐60 bolus cap had excellent conformality to the phantom scalp, with a maximum air gap of 4 mm. TLD measurements showed that the bolus cap generated a uniform dose to the scalp within a 2.7% standard deviation, and the delivered doses agreed with calculated doses to within 2.4% on average. The patient bolus was conformal and the average difference between TLD measured and planned doses was 5.3%.

**Conclusions:**

We have developed a workflow to 3D‐print highly conformal bolus caps for TSI and demonstrated these caps can reproducibly generate a uniform dose to the scalp.

## 1 INTRODUCTION

Total scalp irradiation (TSI) is a specialized treatment technique that aims to deliver a uniform dose to the entire scalp. In the past, TSI has been faced with two major obstacles. First, dose homogeneity, which is substantially limited by the complex field matching required with electron or electron‐photon‐based techniques.^1^ This obstacle has been substantially addressed through transition to using intensity modulation radiation therapy and volumetric modulated arc therapy (VMAT) techniques to eliminate field matching.^2^, ^3^, ^4^, ^5^ The second major obstacle, which still remains, is the need for a scalp bolus in order to ensure adequate dose to the skin.

Making a bolus that is conformal to the scalp is difficult owing to the convex shape of the scalp. Our current standard‐of‐care technique uses sheets of soft 0.5 cm thick commercial bolus material that are cut and taped together and placed on the patient's head to create a bolus cap that is held in place under a swim cap. This method is laborious, time‐consuming, and ultimately produces a bolus cap that is difficult to reproduce for daily treatments and prone to deforming under the swim cap, causing random air gaps. Other bolus fabrication methods for photon‐based TSI presented in the literature suffer from similar limitations. Bedford et al. used an immobilization shell with 1 cm of wax built up on the interior surface. This method suffered from large air gaps between the wax bolus and scalp surface, which led to errors as large as 12% between the planned and delivered dose to the scalp.^2^ Lin et al. used a thermoplastic mesh mask formed to the posterior of the patient's head and then glued 0.5 cm bolus slabs to the surface of the mask. This method had good daily setup reproducibility but still had air gaps as large as 1.5 cm and required the construction of a custom head rest and immobilization device.^5^ Most other TSI studies described in the literature have used 0.5‐ to 1.0‐cm‐thick solid sheets of thermoplastic material that are heated and formed to the patient's medial scalp, with sheets of soft bolus material taped on to cover the lateral portions of the scalp.^4^, ^6^, ^7^ Although these methods have demonstrated good conformality, they still require manual fabrication and are prone to patient discomfort and reproducibility issues.

The use of three‐dimensional (3D) printing to create specialized radiation therapy devices has been a growing area of research. 3D printing offers a minimally labor intensive method to create custom patient‐specific devices using 3D models of patient anatomy that can be derived from CT DICOM data. This has been demonstrated through the use of 3D‐printers in the fabrication of bolus, compensators, and patient‐specific phantoms.^8^, ^9^, ^10^, ^11^, ^12^ Thus far, most 3D‐printed boluses have been used for small and/or relatively flat treatment sites, such as the nose,^11^ ear,^13^ eye canthi,^14^ and foot surface.^10^ Additionally, most 3D‐printed bolus has used standard, rigid thermoplastic materials, including polylactic acid and acrylonitrile butadiene styrene. While these traditional 3D‐printing materials have been shown to be suitable for use as bolus, these materials are unsuitable for a TSI bolus due to the unique challenges associated with TSI. Because the scalp is a relatively extensive treatment area, a bolus made from these materials would be extremely rigid and not practical to fit onto the patient's head in one piece. Additionally, a rigid bolus would be uncomfortable to fit onto a patient and for the patient to wear while in the immobilization setup as TSI patients’ scalps are very sensitive due to radiation‐induced acute skin toxicity.^15^ These issues represent the unique challenges that were considered when developing a 3D printed scalp bolus for TSI. The purpose of this study was to design a 3D‐printed bolus to be used in TSI that improves upon the current problems of nonconformality and limited reproducibility of the bolus cap and that can be readily fabricated as part of a clinical workflow.

## 2 METHODS AND MATERIALS

### 2.A Material analysis

Due to the unique challenges presented in developing a 3D‐printed bolus for TSI, we consulted with a local 3D‐printing company (3D Print Bureau of Texas, Houston, TX, USA) on potential 3D‐printing materials that could be suitable as bolus and meet the requirements of patient comfort for TSI. This company has commercial‐grade PolyJet 3D printers capable of printing materials with many different properties. One such material is Agilus (Stratasys, Eden Prairie, MN, USA), which is a soft‐curing rubber‐like photopolymer resin that can be blended in discrete concentrations with a hard‐curing photopolymer resin during printing to produce objects with varying elasticity ranging from very soft rubber material to a solid block. The ability of 3D‐printed Agilus to produce materials with differing elasticity meant we could select a mixture where the final material closely mimics the flexibility and softness of traditional bolus material. However, the radiological properties of Agilus have not previously been evaluated. Thus, to determine which Agilus mixture would be most appropriate for use as a scalp bolus, we conducted radiological analysis on a spectrum of printed samples with varying mixtures.

Agilus mixtures are characterized by their Shore durometer value. For example, Agilus‐27 has a Shore value of 27 and is the softest material that can be printed, and Agilus‐100 is the firmest material. We first conducted a CT analysis to determine how well the material's physical density was predicted by our standard CT calibration curve. We obtained 25 mm × 200 mm × 5 mm strips printed in the following Shore values: Agilus‐27, Agilus‐40, Agilus‐50, Agilus‐60, and Agilus‐70. The average CT number of each strip was measured using the DICOM imaging software OsiriX (Pixmeo, Bernex, Switzerland) and the clinical CT calibration curve was used to predict the density of each strip. The predicted density was then compared with the true density, which was calculated on the basis of weight (measured with a high‐accuracy scale) and dimensions (measured with calipers).

Of the materials evaluated, Agilus‐60 was identified as the most suitable for a bolus cap (see Section 3) and was therefore further evaluated with percent depth dose (PDD) measurements in 3D printed Agilus‐60 blocks using a method described by Craft and Howell^8^ and briefly summarized here. The external vendor printed blocks of varying sizes with holes for an Exradin A1SL small‐volume ionization chamber. A Varian Truebeam linear accelerator (Varian Medical Systems, Palo Alto, CA, USA) was used to acquire PDD measurements for a 6‐MV beam. A CT scan of the blocks was imported into RayStation, where we modeled the PDD measurement setup in two different ways: one with the density of the blocks overridden with the true measured density of 1.14 g/cm^3^ and one with the density of the blocks derived from the CT calibration curve.

### 2.B Phantom study

A CIRS ATOM anthropomorphic head phantom (CIRS, Norfolk, VA, USA) was used to develop a fabrication workflow and dosimetrically validate the 3D‐printed bolus cap. The head phantom was scanned using a Philips Brilliance Big Bore CT scanner (Philips Healthcare, Andover, MA, USA) using our institution's standard head and neck protocol for CT simulation (3 mm slice thickness, 120 kVp, 400 mAs). The scan was then imported into our commercial treatment planning system (TPS) RayStation 6.99 (RaySearch Laboratories, Stockholm, Sweden).

In the interest of reducing fabrication time to develop an optimal clinical workflow, a Python script (provided in Appendix S1) was developed for RayStation to automatically generate a 5 mm thick patient‐specific bolus cap contour. Before running the script, a rough outline of the desired extent of the bolus cap on the scalp is required. Upon execution, the script generates an external contour of the phantom, and automatically performs the necessary expansions, contractions, and Boolean operations to create the patient‐specific bolus cap contour. Finally, the script uses a built‐in RayStation function to export the bolus cap contour as an .stl file that is compatible with 3D modeling/printing software.

#### 2.B.1 Printing the bolus cap

The Agilus‐60 compound is soft and flexible, like conventional sheets of commercial bolus material, yet is rigid enough to maintain its shape. These properties meant that the bolus cap could be printed in one piece and easily fit onto a patient's head. Because of this, no further modification to the one‐piece bolus .stl file was necessary. The bolus cap was printed in Agilus‐60 by the 3D printing company using a Stratasys PolyJet J750 3D printer.

#### 2.B.2 CT TSI simulation and treatment planning

The 3D‐printed bolus cap was fitted onto the anthropomorphic head phantom and CT scanned using our standard immobilization setup. The head phantom was rested on an Orfit (Orfit Industries, Wijnegem, Belgium) head support and fitted with a three‐point thermoplastic immobilization mask.

The CT scan was imported into RayStation and a VMAT plan was generated using our standard‐of‐care treatment technique: single isocenter, two arcs, 6‐MV photons, and prescription of 60 Gy to 99% of the scalp clinical target volume (CTV) delivered in 30 fractions. The plans were reviewed and approved by a radiation oncologist (A.S.G.) specializing in head and neck treatments.

#### 2.B.3 Dosimetric validation

**Figure 1:**
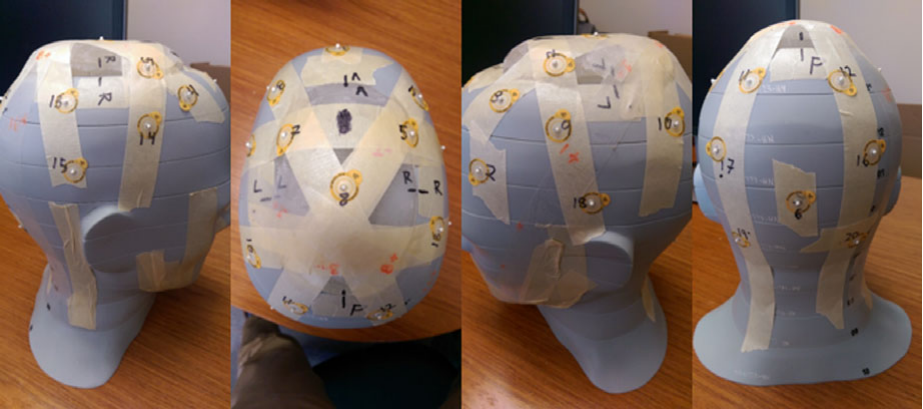
Images of the anthropomorphic head phantom showing the locations of radio‐opaque markers (BBs) used to locate where thermoluminescent dosimeters were placed for dosimetric verification.

To verify that the 3D‐printed bolus cap achieved the necessary surface buildup for a TSI treatment, we performed dosimetric validation using thermoluminescent dosimeters (TLDs). The anthropomorphic head phantom was marked with radio‐opaque markers (BBs) in 20 locations on the scalp within the CTV where TLDs were placed, as shown in Fig. 1. A CT scan of the head phantom with the BBs in place was acquired and registered in the TPS to the primary CT images (used for treatment planning) to mark the locations of the TLDs. The TPS‐calculated dose at the TLD locations was recorded using the average dose within a 0.05 cm^3^ region of interest.

Flat‐pack TLDs (TLD‐100) were taped to the phantom head surface at each of the 20 marked locations. The bolus cap was fitted to the phantom head and placed in the treatment setup. A Varian 21iX linear accelerator was used to deliver one fraction of the planned treatment. Lateral and AP kV images were obtained with the onboard imager to verify the position of the phantom. The TLD were read using a well‐established protocol with a 2.3% uncertainty.^16^

### 2.C Patient study

**Figure 2:**
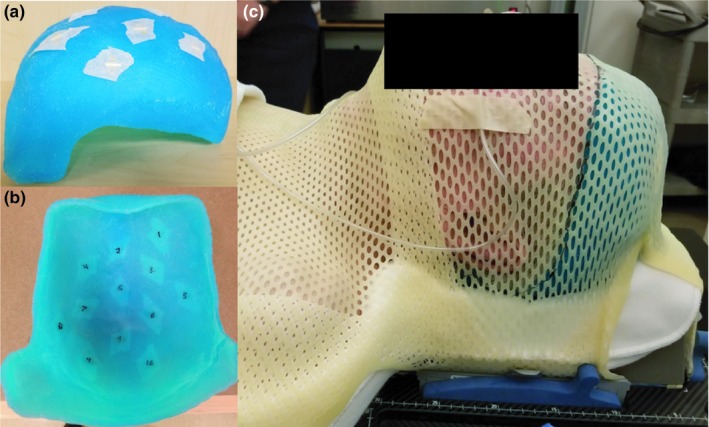
(a) Patient‐specific 3D‐printed Agilus‐60 bolus. (b) Image showing the 12 marked locations for TLD measurements. (c) Image of the CT simulation for the patient study. The location of the bolus cap was marked on the thermoplastic mask to assist in reproducibility of daily setup.

To confirm the results observed in the phantom study translate to real patients and to assess the limits of applicability, a TSI patient was treated using a 3D‐printed scalp bolus generated by the method developed in this study. Our patient was a 78‐year‐old man with squamous cell carcinoma of the scalp with multiple areas of soft tissue nodules and ulceration. The workflow presented in Section 2.B. was used with the patient's head and neck CT simulation scan from a previous treatment to create a 3D model of a 5 mm bolus covering the entire scalp. The bolus was 3D‐printed in Agilus‐60 material by the external printing company and is shown in Fig. 2.

For TSI treatment planning, a CT simulation (standard head and neck protocol) of the patient wearing the 3D‐printed bolus was acquired. The immobilization used consisted of a molded Klarity Head and Shoulder AccuCushion (Klarity Medical Products, Newark, OH, USA) on an Orfit headrest with a 5‐point thermoplastic mask, shown in Fig. 2. An additional 5 mm piece of conventional bolus was abutted to the right side of the bolus cap to cover the patient's right ear and right temple, which the physician wanted to additionally treat. During CT simulation, 12 radio‐opaque markers were affixed on the inside of the bolus cap (shown in Fig. 2) to mark locations for *in vivo* dose measurements with flat‐pack TLDs.

A TSI treatment plan was generated using Pinnacle 9.10 (Philips Healthcare), using the previously described standard‐of‐care VMAT technique with a prescription of 50 Gy to 98% of the scalp PTV (3 mm medial expansion of CTV) delivered in 25 fractions. The TPS‐calculated dose at the marked TLD locations was recorded using the average dose within a 0.05 cm^3^ region of interest. The patient was treated using a Varian TrueBeam linear accelerator. Flat‐pack TLD were affixed both to the locations marked on the inside of the bolus cap and three locations directly on the scalp in the treated area. TLD measurements were acquired on the 2nd, 8th, and 24th fractions of treatment and compared to planned doses.

## 3 RESULTS

### 3.A Material analysis

**Figure 3:**
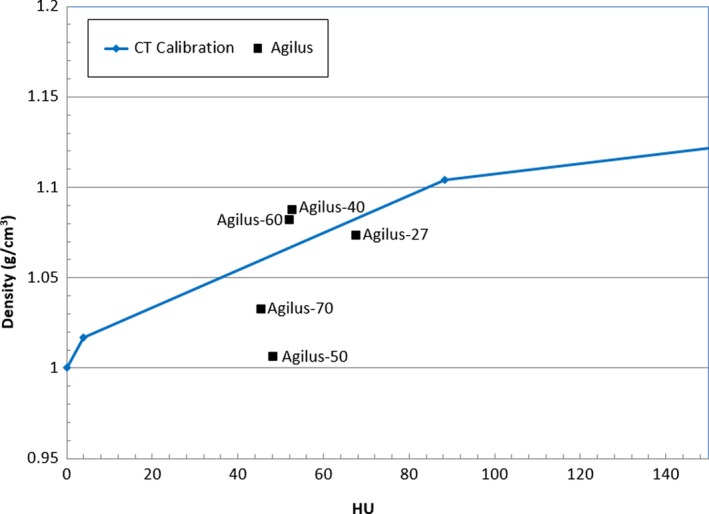
Measured density and CT calibration curve for the different Agilus compound test strips. The squares show the measured density and CT Hounsfield units (HU) for each compound. The line shows the CT calibration curve of the CT scanner used.

The results of the CT analysis and physical measurements of the Agilus compounds are presented in Fig. 3. The Agilus‐27 strip's density was accurately predicted by the CT calibration curve, with a 0.85% error. However, this compound was too deformable, and a test print of an Agilus‐27 bolus cap demonstrated that it could not hold its own shape and would be prone to reproducibility errors. Agilus‐60 was the next best modeled by the CT calibration curve, with a 1.39% error. This material better held its shape, while remaining soft and semi‐flexible, which is why it was chosen for the bolus cap.

**Figure 4:**
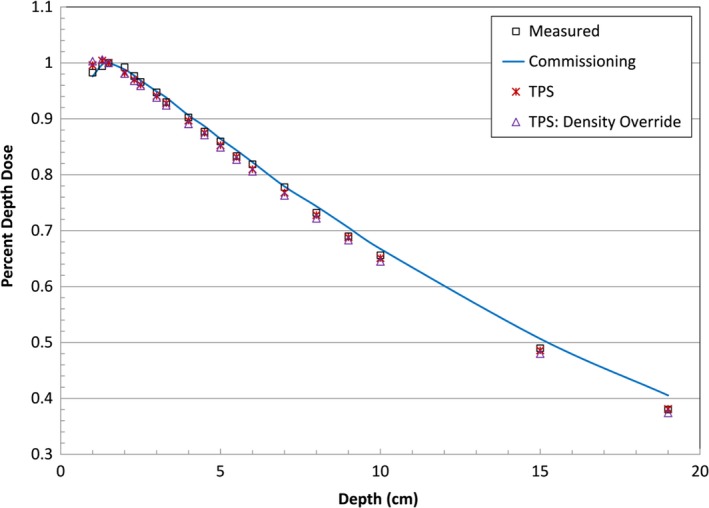
Plot comparing measured PDD in Agilus‐60 (square) with the water commissioning data (line) and TPS modeled PDD with no density override (star) and density overridden to 1.14 g/cm^3^ (triangle).

The results of the PDD measurements are presented in Fig. 4. The water commissioning PDD curve is provided as reference to show the measured PDD behaved similar to water, but deviated at depth due to the higher density of the material. The TPS data agreed very well with the measured data except for points shallower than D_max_, in which the TPS calculated a higher than measured PDD. Overriding the block density in the TPS did not significantly affect the modeled PDD, suggesting that the TPS accurately modeled the heterogeneity correction using our standard CT calibration curve. From the PDD blocks, we found Agilus‐60 to have a density of 1.14 g/cm^3^ and a mean (±SD) HU of 84 ± 33. While Fig. 4 shows the Agilus‐60 strip had a measured density of 1.09 g/cm^3^, this is within expected density variation of 3D‐printed materials.^17^

The CT and PDD measurements demonstrated Agilus‐60 to be a tissue equivalent material suitable for use as bolus.

### 3.B Phantom study

#### 3.B.1 Bolus cap fabrication

**Figure 5:**
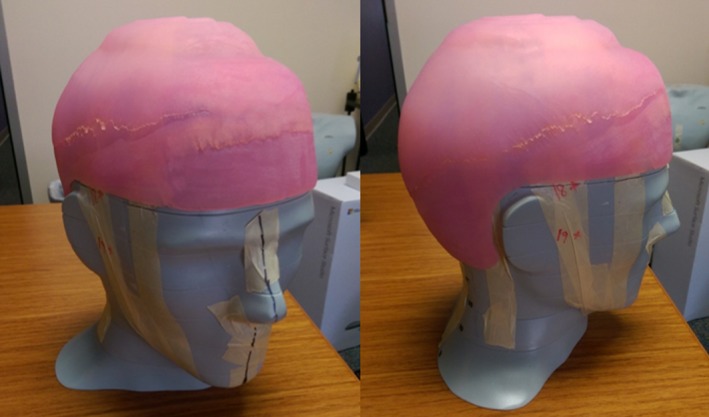
Pictures of the one‐piece Agilus‐60 3D‐printed bolus cap printed by the external company. Note the bolus cap can be printed in any color desired.

Using the Python script we developed, the generation of the bolus cap 3D model .stl file took approximately 10 min. The Agilus‐60 bolus cap printed by the external company took 40 hrs to print and cost $2,381.50, including materials and labor. Pictures of the 3D‐printed bolus cap on the head phantom are shown in Fig. 5.

#### 3.B.2 CT simulation and treatment planning

**Figure 6:**
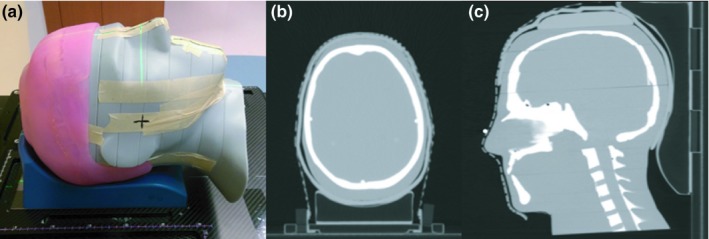
Phantom study computed tomography (CT) simulation. (a) The simulation setup is shown without the mask in place. (b and c) Axial and sagittal CT slices of the Agilus‐60 bolus cap CT simulation.

Photographs of the CT simulation setup and CT images of the bolus cap on the head phantom are presented in Fig. 6. The CT images showed that the bolus cap was conformal to the phantom's scalp, with 4 mm being the maximum air gap observed.

**Figure 7:**
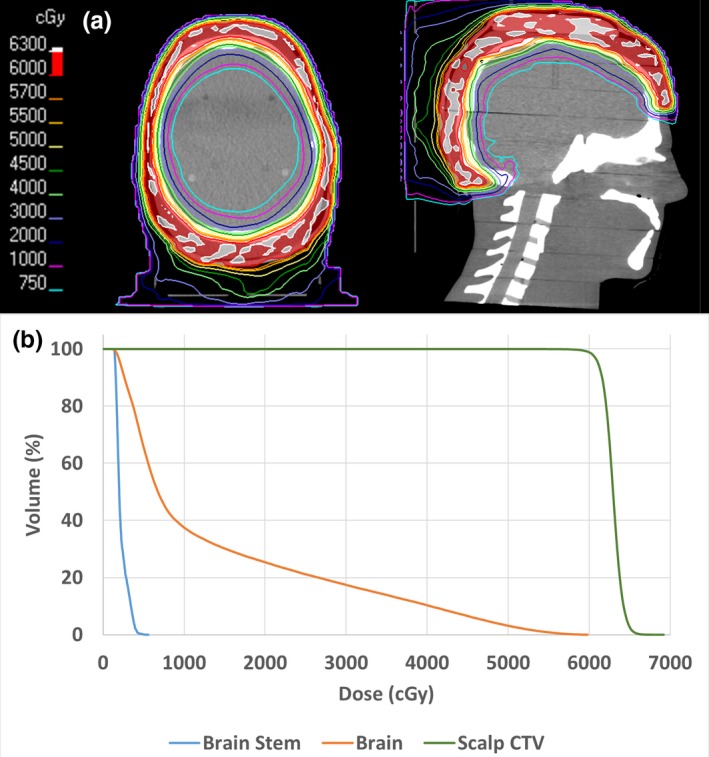
(a) Axial and sagittal isodose distributions for the phantom study. (b) Corresponding dose volume histograms.

Isodose distributions for the VMAT treatment plan generated in RayStation are presented in Fig. 7. The plan achieved the prescription of 60 Gy to 99% of the CTV, with clinically acceptable doses to the brain and brain stem. The white spots seen inside the CTV in the isodose distributions represent 105% hot spots (63 Gy). Hot spots were minimized as much as possible during planning while still maintaining prescription coverage to 99% of the CTV, which is a planning priority and the presence of hot spots to achieve the prescription coverage is an acceptable trade off.

### 3.C Dosimetric validation

Table 1 shows the percent difference between the TPS recorded doses and the TLD measured doses by TLD location (shown in Fig. 2). The 20 TLDs showed the scalp received a mean (±SD) dose of 206.0% ± 2.7% cGy. The average error between the TPS and TLD was 2.4% and the maximum error was 6.3%.

**Table 1:** Absolute percent difference between the TPS calculated and TLD measured dose

TLD location	Percent difference
1	3.4%
2	2.1%
3	2.0%
4	3.1%
5	1.5%
6	1.7%
7	2.4%
8	1.7%
9	1.9%
10	4.6%
11	3.6%
12	1.1%
13	6.3%
14	2.0%
15	3.7%
16	1.0%
17	0.1%
18	1.8%
19	0.5%
20	4.1%
Average	2.4%
SD	1.5%

The average dose of the TLD on the scalp was very close to the prescription dose of 200 cGy, and showed excellent agreement with the TPS calculations (95% of measurements were within 5% of the planned dose). The dosimetric validation results demonstrated that the Agilus‐60 bolus cap generated adequate buildup to treat the scalp surface, and that the dose to the scalp could be accurately calculated by the planning system.

#### 3.C.1 Patient study

**Figure 8:**
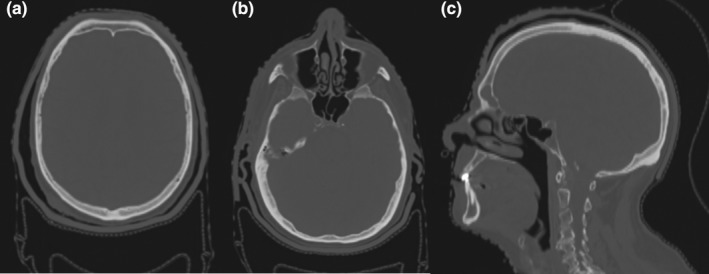
(a) and (b) Axial and (c) sagittal views of the patient's CT simulation with the 3D‐printed Agilus‐60 bolus cap.

The Agilus patient‐specific bolus cap was printed in similar time to that of the phantom bolus cap and cost $1,700. The patient's CT simulation scan showed the bolus to have overall good conformality to the patient's scalp, shown in Fig. 8. The maximum air gap measured was 7 mm, which was larger than observed in the phantom study. The gap was observed on the patient's right side, and may be due to pulling caused by the additional bolus that was added to treat the ear and temple. For future patients with added bolus, conformality could be improved by instructing radiation therapists to better form the thermoplastic mask around the bolus during CT simulation to support the bolus against the scalp.

The patient's TSI treatment plan achieved similar coverage to the phantom study, with the prescribed 50 Gy covering 96% of the scalp CTV and brain V_20_ = 40% and D_1_ = 49.4 Gy.

The average difference between the TLD measured doses on the inside of the bolus and the TPS for the three fractions measured was 5.3% and the maximum error was 9.4%. The interfraction standard deviation averaged 4.2%. Considering the uncertainties inherent in TLD dosimetry and VMAT radiotherapy, these results are within the expected margin of error. These results demonstrate that the bolus was able to be reproducibly setup on the patient as simulated over the course of treatment. The three TLD directly on the scalp recorded an average dose of 204.0 cGy, with all measurements within ±10 cGy of the prescribed dose. This is in good agreement with the results of the phantom study, and confirmed that the bolus generated full build‐up to deliver the prescribed dose to the patient's scalp surface. Additionally, the scalp TLD measurements at the beginning and end of treatment did not show significant differences and recorded full dose over the entire course of treatment. Because the Agilus material is an organic‐based compound, radiation could damage the material through breaking of covalent bonds which can lead to degradation of the physical integrity of the material and negatively affect its build‐up properties.^18^ No significant differences in TLD measured dose over the course of treatment were observed, which demonstrates the Agilus‐60 material did not have any significant degradation in build‐up due to the radiation. Furthermore, the Agilus‐60 bolus cap maintained its initial softness, elasticity, and fit over the entire treatment course, further supporting no significant degradation from radiation.

## 4 DISCUSSION

The technique developed in this study represents a substantial improvement in the fabrication of bolus for TSI. This technique allows for the fabrication of a patient‐specific bolus cap, which proved to have good conformality, with a maximum air gap of only 4 mm in the phantom study and 7 mm when used for a patient. This is a significant improvement compared with the 1–1.5 cm air gaps seen in our existing technique and other techniques presented in the literature.^5^

Agilus‐60 was identified and characterized as a new 3D‐printing material suitable to directly 3D print patient specific boluses or tissue compensators. This material is soft and elastic while being able to remain conformal to complex anatomy, which makes it ideal for use in treatments where patient comfort is a concern. This was a significant factor in our patient study in which the multiple nodules on the patient's scalp were very sensitive. With the soft 3D‐printed bolus, the patient was able to tolerate being in the immobilization setup with the bolus on for the entire course of treatment. Previous studies have used 3D printers to fabricate molds to cast patient‐specific soft silicone boluses.^19^ The Agilus‐60 material and techniques presented in this study offers an improvement over the molding technique as directly 3D printing a soft bolus requires less manual time and labor compared to the time, labor, and material overhead required for molding and casting silicone.

The time reduction for the initial CT simulation of TSI patients enabled by the semi‐automated workflow developed in this study represents one of the major advantages of using a 3D‐printed bolus cap compared with existing bolus techniques. For example, our existing method requires about an hour to fabricate the bolus while the patient is in the CT simulation suite, and physicists, therapists, and a physician must be present. The workflow developed in this study can use a patient's previous diagnostic CT scan to create the bolus cap 3D model, which allows for the bolus cap to be fabricated in advance of the patient's CT simulation. This vastly reduces the time required for a TSI simulation to only ~20 min to set up patient immobilization, which lowers the time commitment of the patient and frees up valuable staff time. The radiation therapists also voiced their preference for the 3D‐printed bolus compared to our existing technique as the one piece 3D‐printed bolus was more reproducible for daily treatment than our existing method, which was prone to falling apart and having to be repaired several times over the course of treatment. Comparatively, the 3D‐printed bolus was faster and easier to setup for daily treatment as it maintained its shape. The main time limitation of this workflow is the time required to print the bolus, but it is important to note that this is not staff‐involved time and the bolus can be printed overnight. Also, it is relevant to point out that this was designed to be a single planning system workflow, that is, with both bolus cap design and VMAT treatment planning fully carried‐out in RayStation; this is currently being implemented. However, at the time of the patient case described here, we were still in the process of transitioning all clinical planning from the Pinnacle to RayStation TPS.

Future research will include using 3D‐printed Agilus bolus for other complex anatomy requiring bolus such as soft‐tissue sarcomas and generalizing the python script to be compatible with creating bolus for these applications.

In conclusion, we developed a semi‐automated workflow for the fabrication of highly conformal, patient‐specific 3D‐printed bolus caps for use in TSI. Material analysis identified Agilus‐60 as a new 3D‐printing material with suitable physical and radiological properties for use as a bolus in radiation therapy. An end‐to‐end phantom study demonstrated that the fabrication method developed created a conformal bolus and subsequent dosimetric validation measurements demonstrated that the 3D‐printed bolus cap generated a uniform dose to the scalp that could be accurately calculated by the TPS, and therefore met the clinical requirements for TSI. A patient study showed the technique worked well with a patient and the bolus cap reproducibly delivered full dose to the scalp. Additionally, the technique offered significant advantages to our clinical workflow for TSI.

## Supporting information


**Appendix S1.** RayStation Python script for automated generation of bolus cap .stl model file.
