# Supplementary material for: Development and validation of a 3D‐printed bolus cap for total scalp irradiation
Source: J Appl Clin Med Phys. 2019 Mar 1;20(3):89–96. doi: 10.1002/acm2.12552 (PMC6414136; doi:10.1002/acm2.12552)
Supplement: Supplementary file 1 — Appendix S1. RayStation Python script for automated generation of bolus cap .stl model file. [file ACM2-20-89-s001.docx]

**Appendix 1**

# Script to create a patient-specific total scalp bolus contour using patient's CT

# This script is under The MIT License (MIT) Copyright (c) 2018 Garrett Baltz

# INSTRUCTIONS:

# This script requires an ROI named "Bolus_Outline"

# Bolus_Outline should be drawn along the sagittal plane and encompass the desired extent of the scalp bolus

# The contour should first be drawn on the center sagittal slice of the CT.

# The contour should then be copied to a slice where the ears are visible so they can be contoured out

# Copy this contour to replace the original center contour, and also paste the contour on the opposite side of the patient

# Interpolate the contour so it extends across all sagittal slices of the patient

# Once this contour is in place, the script can be run.

# The script will save the final bolus model as an .STL file to the directory specified

from connect import *

import sys

import clr

clr.AddReference("System.Windows.Forms")

clr.AddReference("System.Drawing")

from System.Windows.Forms import Application, Form, Label, TextBox, Button, MessageBox

from System.Drawing import Point, Size

# Load current patient

patient = get_current('Patient')

case = get_current("Case")

examination = get_current("Examination")

# Load in names of all ROIs

roi_names = [r.Name for r in case.PatientModel.RegionsOfInterest]

# Check if Bolus Outline has been defined, if not tell user and exit script

if 'Bolus_Outline' not in roi_names:

await_user_input('Bolus_Outline ROI does not exist! Please create then continue script')

# Generate External body contour

with CompositeAction('Create external (BodyROI)'):

retval_0 = case.PatientModel.CreateRoi(Name="BodyROI", Color="0, 128, 64", Type="External", TissueName="", RoiMaterial=None)

retval_0.CreateExternalGeometry(Examination=examination, ThresholdLevel=-250)

# prompt user to check that external ROI is correct and doesn't include couch/headrest

await_user_input('Review the created external contour and edit if necessary')

# Create the expanded body contour

with CompositeAction('Expand (BodyROI)'):

retval_1 = case.PatientModel.CreateRoi(Name="Bolus", Color="Yellow", Type="Bolus", TissueName=None, RoiMaterial=None)

retval_1.SetMarginExpression(SourceRoiName="BodyROI", MarginSettings={ 'Type': "Expand", 'Superior': 0.5, 'Inferior': 0.5, 'Anterior': 0.5, 'Posterior': 0.5, 'Right': 0.5, 'Left': 0.5 })

retval_1.UpdateDerivedGeometry(Examination=examination, Algorithm="Auto")

# subtract contour

with CompositeAction('ROI Algebra (BolusOutline2)'):

retval_2 = case.PatientModel.CreateRoi(Name="BolusOutline2", Color="Blue", Type="Organ", TissueName=None, RoiMaterial=None)

retval_2.SetAlgebraExpression(ExpressionA={ 'Operation': "Intersection", 'SourceRoiNames': ["Bolus"], 'MarginSettings': { 'Type': "Expand", 'Superior': 0, 'Inferior': 0, 'Anterior': 0, 'Posterior': 0, 'Right': 0, 'Left': 0 } }, ExpressionB={ 'Operation': "Intersection", 'SourceRoiNames': ["Bolus_Outline"], 'MarginSettings': { 'Type': "Expand", 'Superior': 0, 'Inferior': 0, 'Anterior': 0, 'Posterior': 0, 'Right': 0, 'Left': 0 } }, ResultOperation="Intersection", ResultMarginSettings={ 'Type': "Expand", 'Superior': 0, 'Inferior': 0, 'Anterior': 0, 'Posterior': 0, 'Right': 0, 'Left': 0 })

retval_2.UpdateDerivedGeometry(Examination=examination, Algorithm="Auto")

# create final bolus contour

with CompositeAction('ROI Algebra (FinalBolus)'):

retval_3 = case.PatientModel.CreateRoi(Name="FinalBolus", Color="White", Type="Organ", TissueName=None, RoiMaterial=None)

retval_3.SetAlgebraExpression(ExpressionA={ 'Operation': "Union", 'SourceRoiNames': ["BolusOutline2"], 'MarginSettings': { 'Type': "Expand", 'Superior': 0, 'Inferior': 0, 'Anterior': 0, 'Posterior': 0, 'Right': 0, 'Left': 0 } }, ExpressionB={ 'Operation': "Union", 'SourceRoiNames': ["BodyROI"], 'MarginSettings': { 'Type': "Expand", 'Superior': 0, 'Inferior': 0, 'Anterior': 0, 'Posterior': 0, 'Right': 0, 'Left': 0 } }, ResultOperation="Subtraction", ResultMarginSettings={ 'Type': "Expand", 'Superior': 0, 'Inferior': 0, 'Anterior': 0, 'Posterior': 0, 'Right': 0, 'Left': 0 })

retval_3.UpdateDerivedGeometry(Examination=examination, Algorithm="Auto")

# Underive ROIs

if case.PatientModel.RegionsOfInterest['Bolus'].DerivedRoiExpression:

case.PatientModel.RegionsOfInterest['Bolus'].DeleteExpression()

if case.PatientModel.RegionsOfInterest['BolusOutline2'].DerivedRoiExpression:

case.PatientModel.RegionsOfInterest['BolusOutline2'].DeleteExpression()

if case.PatientModel.RegionsOfInterest['FinalBolus'].DerivedRoiExpression:

case.PatientModel.RegionsOfInterest['FinalBolus'].DeleteExpression()

# Delete helper intermediary ROIs

case.PatientModel.RegionsOfInterest['BolusOutline2'].DeleteRoi()

case.PatientModel.RegionsOfInterest['Bolus'].DeleteRoi()

# pause script and prompt user to review bolus contour

await_user_input('Review the final bolus contour and edit if necessary (check around ears)')

#Define Forms class that will tell the user when script has finished and where stl file is saved

class SelectROIForm(Form):

def __init__(self):

# Set the size of the form

self.Size = Size(500, 200)

# Set title of the form

self.Text = 'Enter Save Directory'

# Add a label

label = Label()

label.Text = 'Enter save directory path below:'

label.Location = Point(15, 15)

label.AutoSize = True

self.Controls.Add(label)

label2 = Label()

label2.Text = 'Directory needs to be in a network drive (e.g. Y: or S:)'

label2.Location = Point(15, 30)

label2.AutoSize = True

self.Controls.Add(label2)

#Add textbox

self.textbox = TextBox()

self.textbox.Text = 'S:/SHARED/Radiation physics/'

self.textbox.Location = Point(15, 60)

self.textbox.Size = Size(300, 20)

self.Controls.Add(self.textbox)

# Add button to press OK and close the form

button = Button()

button.Text = 'OK'

button.AutoSize = True

button.Location = Point(15, 100)

button.Click += self.ok_button_clicked

self.Controls.Add(button)

def ok_button_clicked(self, sender, event):

# Method invoked when the button is clicked

# Save the entered directory name

self.save_dir = self.textbox.Text

# Close the form

self.Close()

# Create an instance of the form and run it

form = SelectROIForm()

Application.Run(form)

# save before export

patient.Save()

#Export Final Bolus to user's network drive

case.PatientModel.StructureSets[examination.Name].RoiGeometries['FinalBolus'].ExportRoiGeometryAsSTL(DestinationDirectory=form.save_dir, OutputUnit ='Millimeter')
